# Supplementary material for: Bayesian active sound localisation: To what extent do humans perform like an ideal-observer?
Source: PLoS Comput Biol. 2025 Jan 7;21(1):e1012108. doi: 10.1371/journal.pcbi.1012108 (PMC11741579; doi:10.1371/journal.pcbi.1012108)
Supplement: Attachment — Submitted filename: SS_Insights into dynamic sound localisation.docx [file pcbi.1012108.s001.docx]

Insights into dynamic sound localisation: A direction-dependent comparison between human listeners and a Bayesian model

**Summary** –

The study aims to model the contribution of head movements in sound localization using a Bayesian approach and validate this model with behavioral data collected from virtual reality or free-field environments. However, the research question lacks clarity and focus, which significantly undermines the potential value of the findings. Upon reviewing the paper, I encountered numerous issues, both textual and conceptual. Unfortunately, the problems are too extensive to address individually or line by line.

Title – ‘Insights into dynamic sound localisation’ What are the insights?

Abstract – It is unclear what the authors mean by ‘dynamic sound localization’? Do the authors refer to dynamic as if the sound is either moving, changing over space and or time, amplitude or frequency?

Numerous studies have demonstrated that accurate sound localization of brief, stationary sounds is possible without head movement. While head movements are known to be effective in resolving front-back confusions, the the study does not provide convincing mechanisms for how head movements might enhance overall localization accuracy beyond this specific benefit.

The abstract lacks clarity regarding the measurement of sound localization, as the methods section indicates a combination of virtual reality and free-field environments. It woul be good to highlight that in the abstract.

The alignment between the behavioral data and the proposed model is questionable. This discrepancy is particularly evident in the case of the 500 ms duration measurements, which show no correlation with the model's predictions. The limited scope of the behavioral data, collected for only a single duration, further undermines the model's validity and generalizability.

**Introduction**

Line 6-7 –“ Dynamic acoustic cues …reverberant or virtual environments”

The study does not test reverberant environments, which raises questions about its mentioning here. In the tested paradigm, one would expect the spectral cues to remain intact. Previous research has shown that randomizing spectral cues can lead to the use of binaural cues, particularly interaural level differences. However, this aspect is not the focus of the current study.

Lines 10 – 16 – It is mentioned that head movements should improve the localization accuracy. Why would that be apart from resolving front-back or up-down confusion?

It is has been shown that for azimuth even short noise bursts (10-50ms) are highly localizable and 100 ms. for elevation (Vliegen 2004). What additional information does head movement provide. This is one of my major points that I think need to be addressed to formulate a robust hypothesis.

Lines 19 -21 ’Due to their complicated methodology’ I would argue that it possible that the head movement does not explicitly provide any additional cue and therefore there are very limited studies investigating it. It is not necessarily a complex method because sound localization with head movements as response measure has been around for 3-4 decades.

**Methods**

Model

I am quite content with the model description and implementation. I could follow the model reasonably and agree with assumptions around the priors.

Behavior experiments

I am highly confused on why Virtual reality (VR) head-mounted display was used during the experiment.

Lines 227 – ‘visual representation’ It is totally unclear on what kind of visual information was available and why?

The presentation of the experimental setup is confusing and potentially problematic. Initially, it appears that the localization task was performed in virtual reality (VR). However, it is only revealed later that the sounds were actually presented in a free-field environment. This lack of clarity raises significant concerns about the methodology. More critically, the use of a VR headset during free-field sound presentation is troubling. The VR headset, being a substantial object on the participant's face, is likely to interfere with and distort the spectral cues of the sounds presented through speakers in the free-field environment. This interference could significantly impact the validity of the localization data collected. This potential perturbation of spectral cues due to the presence of the VR headset is a major methodological issue that the authors do not appear to address. It could introduce uncontrolled variables and potentially compromise the integrity of the study's results, especially given the importance of spectral cues in sound localization.

Lines 249 – Why was vector based panning amplitude used to present point or single sound sources? I wonder if the experimental setup would allow to play a single sound from a single speaker, then why not use that methodology?

**Procedure**

Why was eventually hand tracking used to register the response? Earlier head movements from the head-trackers of the VR are used. I believe same could be used to register the response in a more natural way of orienting towards sounds.

It is not easy to follow how long the stimulus was in each trial? A graphic representation of the trial length might help to bring clarity.

**Results**

I have an objection regarding the behavioral dataset for 500ms while the model is for only 100 ms.

Table 1

The errors in elevation (polar RMSE) are quite high and makes wonder again if the VR interferes with the incoming sounds and perturbs the spectral cues. Other studies report errors in elevation response around 10-15 degrees (Markous & Middlebrooks 1991; Carlie et al., 1997).

The authors central argument that head movements should reduce localization errors appears to be contradicted by their own results. Upon examination of both the model predictions and the behavioral data, there is no discernible difference in response errors between static and dynamic conditions. This lack of difference stands in direct opposition to the authors' postulation regarding the beneficial effects of head movements on sound localization accuracy. This discrepancy between the authors' theoretical framework and their empirical findings raises significant questions about the validity of their hypothesis and the overall conclusions of the study. It suggests a fundamental misalignment between the proposed theory and the observed data, which undermines the core premise of the research. The failure to demonstrate a reduction in localization errors with head movements, as predicted by their model, calls into question the utility of the proposed Bayesian model and the purported advantages of dynamic sound localization strategies. This inconsistency should have been addressed and explained by the authors, as it represents a critical weakness in their argument.

A recommendation for the authors is to include the raw localization responses in their presentation of data, prior to introducing group data and statistics. This addition would significantly enhance the clarity of both the tasks performed and the statistical analyses employed.

Lines 308 -312 : The authors discuss that the yaw should in principle not reduce up-down confusion and allude to possible roll component. This is likely the cause, but it would be informative to quantify this as the authors probably have the data from the head tracker.

Effect of stimulus duration – Fig3.

As I mentioned earlier, I see two problems here. One that the behavioral data presented is only for the duration of 500 ms. Second, the behavioral data doesn’t correlate with the model prediction.

Lines 439-442: The authors do acknowledge the difference in the model and the data. However, there is no direct explanation on what might account for these differences rendering doubts on the reliability of the model.

**Conclusion**
In its current form, the paper is difficult to follow and leaves too many unanswered questions about its methodologies, results, and overall significance. A major revision is required to clarify the research questions, streamline the introduction, refine the methodology, and provide a comprehensive and comprehensible presentation of the results. However, the potential for publication after these major revisions remains uncertain.
