# Supplementary material for: Bayesian active sound localisation: To what extent do humans perform like an ideal-observer?
Source: PLoS Comput Biol. 2025 Jan 7;21(1):e1012108. doi: 10.1371/journal.pcbi.1012108 (PMC11741579; doi:10.1371/journal.pcbi.1012108)
Supplement: Attachment — Submitted filename: PLOS response.docx [file pcbi.1012108.s002.docx]

**General reply to the editor and reviewers**

Thank you for taking your time to read our manuscript and provide useful improvements. Based on your comments, we implemented two large-scale changes:

1. We have adapted our model to be able to simulate the **exact** head movements that were made by the participants. This means that the duration and rotation axes of each trial are now the same in both the model and behavioural data.
2. We streamlined the paper’s research goal, focusing on the question "to what extent can human active sound localisation be predicted by an ideal-observer model?" Accordingly, our results now focus on the differences between the model and behavioural data, and the discussion on potential reasons for any discrepancy we have found.

In the following, we respond to your comments line-by-line, with our responses marked with bullet points.

**Reviewer 1**

The study aims to model the contribution of head movements in sound localization using a Bayesian approach and validate this model with behavioral data collected from virtual reality or free-field environments. However, the research question lacks clarity and focus, which significantly undermines the potential value of the findings. Upon reviewing the paper, I encountered numerous issues, both textual and conceptual. Unfortunately, the problems are too extensive to address individually or line by line.

- We thank the reviewer for their extensive feedback. We feel that it has helped us to improve the clarity and quality in several aspects of the manuscript.

Title – ‘Insights into dynamic sound localisation’ What are the insights?

- We agree that the title does not properly convey the purpose of the study. An adjustment was made.

Abstract – It is unclear what the authors mean by ‘dynamic sound localization’? Do the authors refer to dynamic as if the sound is either moving, changing over space and or time, amplitude or frequency?

- We agree about this ambiguity. Because of this, we have changed the term "dynamic localisation" throughout the paper to "active localisation". This emphasizes that all changes in cues are due to active head movements by the listener.

Numerous studies have demonstrated that accurate sound localization of brief, stationary sounds is possible without head movement. While head movements are known to be effective in resolving front-back confusions, the study does not provide convincing mechanisms for how head movements might enhance overall localization accuracy beyond this specific benefit.

- The benefits of head movements become apparent when spectral cues are absent or distorted. However, the goal of our work was not to emphasise the importance of head movements, this has been done in a plethora of studies in the past. The goal of our work was to investigate to what extent human performance can be predicted by an optimal Bayesian observer. We agree that this might have been not clearly stated in the previous manuscript version. Thus, now, we added a sentence at the end of the introduction more specifically describing this goal. Note that our direction-dependent analysis also aims to show how head movements affect different source directions differently. On the other hand, sources with uninformative spectra were not tested in the present paper, as we intended to validate the model under the most elementary conditions first before moving onto more complex situations, which is unfortunately outside the scope of the manuscript.

The abstract lacks clarity regarding the measurement of sound localization, as the methods section indicates a combination of virtual reality and free-field environments. It would be good to highlight that in the abstract.

- Thanks for the pointer – we clarified that. We hope to be more clear that virtual reality was only applied by means of a head-mounted display (HMD) used for head tracking, visual feedback of the responses, and hiding of the loudspeakers without disorienting the subject (in Majdak et al. 2010 "3-D localization of virtual sound sources: Effects of visual environment, pointing method, and training", localisation precision was found to be significantly worse in dark rooms compared to using an HMD). While HMDs indeed may affect the listener's HRTFs, these differences are usually not significantly affecting localisation performance (Poirier-Quinot & Lawless, 2023, “Impact of wearing a head-mounted display on localization accuracy of real sound sources.”) Note that our subjects **did not wear** headphones and sounds were **played over loudspeakers**. We now also have clarified that in the manuscript.

The alignment between the behavioral data and the proposed model is questionable. This discrepancy is particularly evident in the case of the 500 ms duration measurements, which show no correlation with the model's predictions. The limited scope of the behavioral data, collected for only a single duration, further undermines the model's validity and generalizability.

- We agree that the discrepancy in tested durations undermined the validity of the comparison between experimental and model results. Hence, we adjusted the model trial durations to match those of the behavioural experiment. The discrepancy found earlier was resolved by removing the dynamic spectral cue from the model, based on the findings of Mclachlan et al. 2023 "Dynamic spectral cues do not affect human sound localization during small head movements." Therefore, now only dynamic ITD is used in addition to the static cues, making the model to perform much more similar to human listeners. We now describe these changes in the manuscript.

**Introduction**

Line 6-7 –“ Dynamic acoustic cues …reverberant or virtual environments”

The study does not test reverberant environments, which raises questions about its mentioning here. In the tested paradigm, one would expect the spectral cues to remain intact. Previous research has shown that randomizing spectral cues can lead to the use of binaural cues, particularly interaural level differences. However, this aspect is not the focus of the current study.

- We agree that this might be an interesting additional condition to test our model. However, currently this seems to be outside of the scope of our study, which introduces a model that can be tested under variety of interesting conditions in future studies. A small section about further research was added to the conclusion.
- That sentence cited from our Introduction aimed to explain the context under which dynamic cues are the most important. We used broadband noise for better comparability with studies that used the same stimulus type such as Middlebrooks (1999, “Virtual localization improved by scaling nonindividualized external-ear transfer functions in frequency”) and Carlile et al (1997, “The nature and distribution of errors in sound localization by human listeners”).

Lines 10 – 16 – It is mentioned that head movements should improve the localization accuracy. Why would that be apart from resolving front-back or up-down confusion?

- Elevation estimation can improve. Theoretically, the rate of change in binaural cues when rotating the head can be informative to estimate the source elevation. For yaw rotation, binaural cues change faster for sources on the horizon and don't change at all for sources above the listener, thus, the rate of change in ITD can be an informative cue. It has been termed the ‘Wallach cue’ in Perrett & Noble (1997, “The contribution of head motion cues to localization of low-pass noise.”) and also more extensively tested in Jiang et al (2019, ”The role of dynamic cue in auditory vertical localisation”). The paper by Perrett & Noble shows that sources with little spectral information (low-pass noise) have significantly improved elevation estimation if movement is allowed. This has been added to the manuscript introduction.

It has been shown that for azimuth even short noise bursts (10-50ms) are highly localizable and 100 ms. for elevation (Vliegen 2004). What additional information does head movement provide. This is one of my major points that I think need to be addressed to formulate a robust hypothesis.

- For azimuth, we fully agree. We hope to be clear in the manuscript, avoiding any claims that head movement may improve azimuth estimation.
- For elevation, there is existing evidence that head movements help, especially when spectral cues are distorted.

Lines 19 -21 ’Due to their complicated methodology’ I would argue that it is possible that the head movement does not explicitly provide any additional cue and therefore there are very limited studies investigating it. It is not necessarily a complex method because sound localization with head movements as response measure has been around for 3-4 decades.

- We agree – that was a misleading formulation. We rewrote the corresponding paragraphs, hoping to qualify our statements and being more clear on the aims of our study.

**Methods**

Model

I am quite content with the model description and implementation. I could follow the model reasonably and agree with assumptions around the priors.

Behavior experiments

I am highly confused on why Virtual reality (VR) head-mounted display was used during the experiment.

Lines 227 – ‘visual representation’ It is totally unclear on what kind of visual information was available and why?

The presentation of the experimental setup is confusing and potentially problematic. Initially, it appears that the localization task was performed in virtual reality (VR). However, it is only revealed later that the sounds were actually presented in a free-field environment. This lack of clarity raises significant concerns about the methodology. More critically, the use of a VR headset during free-field sound presentation is troubling. The VR headset, being a substantial object on the participant’s face, is likely to interfere with and distort the spectral cues of the sounds presented through speakers in the free-field environment. This interference could significantly impact the validity of the localization data collected. This potential perturbation of spectral cues due to the presence of the VR headset is a major methodological issue that the authors do not appear to address. It could introduce uncontrolled variables and potentially compromise the integrity of the study’s results, especially given the importance of spectral cues in sound localization.

- It appears that the description in our methodology has caused some confusion, and we have now adjusted it accordingly. The head-mounted display (HMD) was used for three reasons: 1) The subject’s head movements needed to be tracked, 2) The subject needed visual feedback on their orientation in space without seeing the loudspeakers 3) The subject needed visual feedback on their responses.

  Note that similar conditions could have been accomplished in a dark room, with a laser pointer and a separate head tracker, but the HMD solved all problems at once. Furthermore, localisation precision seems to be better with an HMD than in a dark room, likely because subjects get disoriented without any visual information about their orientation (Majdak et al, 2010, “3-D localization of virtual sound sources: Effects of visual environment, pointing method, and training.”).

  Note also that the HRTFs were measured without the HMD, whereas the spectral cues during the experiment are indeed partially affected by an HMD. However, these differences were found to only minimally affect localisation performance (Poirier-Quinot & Lawless, 2023, “Impact of wearing a head-mounted display on localization accuracy of real sound sources” and Gupta et al., 2018, “Investigation of effect of VR/AR headgear on Head related transfer functions for natural listening”).

  We now have added this reasoning to the Methods section.

Lines 249 – Why was vector based panning amplitude used to present point or single sound sources? I wonder if the experimental setup would allow to play a single sound from a single speaker, then why not use that methodology?

- The distribution of loudspeakers in the array did not allow for testing at the desired angles, e.g., it was not possible to test loudspeakers at the same lateral angle along different polar angles.

  The elevation span between loudspeakers in the array was 10 degrees at most. The increase in polar error was close to zero for a loudspeaker span of 10 degrees using VBAP (Baumgartner & Majdak, 2015, “Modeling localization of amplitude-panned virtual sources in sagittal planes”). Also, the polar precision of listeners was expected to be below that elevation span, see Bremen, P., van Wanrooij, M. M., & van Opstal, A. J. (2010). “Pinna cues determine orienting response modes to synchronous sounds in elevation.”. For these reasons, we do not see any evidence that the use of VBAP in our experiment had any substantial effect on listeners’ localisation performance.

**Procedure**

Why was eventually hand tracking used to register the response? Earlier head movements from the head-trackers of the VR are used. I believe same could be used to register the response in a more natural way of orienting towards sounds.

- Head movements were made regardless of the source direction, it was always a 10 degree rotation to the left or to the right. Head tracking would restrict responses to the frontal hemisphere, furthermore earlier studies show that hand pointing and head pointing do not result in statistically different responses in the frontal hemisphere (see Majdak et al.(2010). “3-D localization of virtual sound sources: Effects of visual environment, pointing method, and training.” Or Haber et al. (1993) “Comparison of nine methods of indicating the direction to objects: data from blind adults.”) Visual feedback of the hand pointer was provided in VR.

It is not easy to follow how long the stimulus was in each trial? A graphic of the trial length might help to bring clarity.

- In the static condition, the stimulus duration was 500 ms.
- In the dynamic condition, the stimulus duration depended on the subject’s rotation speed because the stimulus ended when the subject has rotated the head by 10 degrees.
- We have discussed various ways of graphical representation of the stimulus duration. While we finally ended up at refraining from having an additional figure in the manuscript, we now provide statistics (mean and SD) of the stimulus durations for the dynamic condition. And better explain the stimulus duration in text.

**Results**

I have an objection regarding the behavioral dataset for 500ms while the model is for only 100 ms.

- We agree and have adapted the approach accordingly.

Table 1

The errors in elevation (polar RMSE) are quite high and makes one wonder again if the VR interferes with the incoming sounds and perturbs the spectral cues. Other studies report errors in elevation response around 10-15 degrees (Markous & Middlebrooks 1991; Carlie et al., 1997).

- We fully agree that there is a discrepancy. This is because localisation errors are direction dependent, e.g., Makous & Middlebrooks (1991) only tested up to 45 degrees elevation, Carlile et al. (1997) only tested up to 40 degrees, whereas we have tested up to 90 degrees. The largest errors in our study were found at directions above the listener, yielding a larger average error. However, note that the metrics are different: we used the RMSE, whereas Makous & Middlebrooks (1991) and Carlie et al., (1997) used the standard deviation and mean bias. Given that, we think that our results do in fact agree with similar studies. We now summarise that in Tab 2.

The authors central argument that head movements should reduce localization errors appears to be contradicted by their own results. Upon examination of both the model predictions and the behavioral data, there is no discernible difference in response errors between static and dynamic conditions. This lack of difference stands in direct opposition to the authors’ postulation regarding the beneficial effects of head movements on sound localization accuracy. This discrepancy between the authors’ theoretical framework and their empirical findings raises significant questions about the validity of their hypothesis and the overall conclusions of the study. It suggests a fundamental misalignment between the proposed theory and the observed data, which undermines the core premise of the research. The failure to demonstrate a reduction in localization errors with head movements, as predicted by their model, calls into question the utility of the proposed Bayesian model and the purported advantages of dynamic sound localization strategies. This inconsistency should have been addressed and explained by the authors, as it represents a critical weakness in their argument.

- As it seems, we were not clear about the purpose of our study. Now, we have made an effort to resolve this confusion. Our goal was not to discuss the effects of head movement, but rather to investigate whether humans optimally utilize acoustic and sensorimotor cues when performing the task of sound localization. For example, our model predicted significant improvement in polar RMSE but humans did not show this improvement. In the discussion, we elaborate on the reasons of this discrepancy. While behavioural experiments show that head movements further improve elevation estimation with distorted spectral information, such conditions are a natural next step for testing. Note that such tests would require the Bayesian model to have been tested under the simplest conditions first and this is the goal of our study.
- We note that this aspect is very important to understand the impact of our study, thus, we have added this to the end of the Discussion section.

A recommendation for the authors is to include the raw localization responses in their presentation of data, prior to introducing group data and statistics. This addition would significantly enhance the clarity of both the tasks performed and the statistical analyses employed.

- Thanks for the pointer. We now provide an example showing how raw localisation responses have been translated into Kent distributions.

Lines 308 -312 : The authors discuss that the yaw should in principle not reduce up-down confusion and allude to possible roll component. This is likely the cause, but it would be informative to quantify this as the authors probably have the data from the head tracker.

- Thanks for the hint - we followed your advice and adapted the model to be able to exactly follow the actual data from the head tracker.
- Now, given that the model follows the same rotations as the subjects did, we see the same reduction in up-down confusions. Note that the tracker statistics showed that only small roll movements were made on average. We added this information in the manuscript, among the other explanation that the Wallach cue improved elevation estimation, which reduced the edge cases that crossed the horizontal plane and resulted in an up-down confusion.

Effect of stimulus duration – Fig3.

As I mentioned earlier, I see two problems here. One that the behavioral data presented is only for the duration of 500 ms. Second, the behavioral data doesn’t correlate with the model prediction.

- We agree. With all the changes we did for this revision, we hope that these two problems have been resolved.

Lines 439-442: The authors do acknowledge the difference in the model and the data. However, there is no direct explanation on what might account for these differences rendering doubts on the reliability of the model.

- The duration section has been removed due to the fact that the model now follows the exact movements from the experiment. The decision was made to omit dynamic spectral cues, with the result that the model output now matches the experimental data much better (see above).  The duration section was replaced with a section that investigates the influence of the update rate, i.e., the time step size, of the model.

**Reviewer 2**

272) A qualitative description of how the model does and does not operate would be helpful especially for readers unfamiliar with the authors’ previous paper introducing the model. By this I mean in particular that it should be made explicit that the model uses motion implicitly and does not actually calculate any dynamic cues (such as the sign or magnitude of the ratio of delta_ITD to delta_yaw). The templates contain only static cues associated with each location in head-centred coordinates, and (as I understand it) motion essentially serves to eliminate static ambiguities (e.g. ITD-based cone-of-confusion and possible front/back spectral cue similarities) by continually re-registering the head-centred coordinates with world-centred (or torso- or zero-yaw-centred) ones to allow template similarity to “pile up” near the veridical source location over multiple temporal snapshots.

- We agree. We also thank for correctly describing the model’s conceptual framework.
- We have now added a similar qualitative description of it in the manuscript introduction. We also agree that it would be very interesting to consider dynamic cues as separate cues, instead of a series of static snapshots. However, we are not aware of concrete physiological evidence that humans have such an auditory counterpart to the “velocity” detector found in the eye.

Additionally, it would be helpful to add a figure showing how the posterior probability distribution develops over the course of a simulated head-turn, eliminating erroneous location modes in the PDF.

- We agree that this makes the processing in the model a lot more intuitive. We added a figure.

2) Clarification of the relationship between the conditions of simulation and the behavioural experiment is needed. This caused me considerable confusion on first reading because I was expecting more congruency between the conditions. In the simulation, the head executes a yaw starting at rest at 0 degrees with an acceleration of 20 deg/s/s. The simulation is then run over varying durations (5-1000 ms) of that motion, which results in different amounts of total yaw, depending on duration (0.1 degrees for 100 ms, 2.5 degrees for 500 ms, 10 degrees for 1000 ms), although this is not emphasized in the text. In contrast, in the behavioural experiment, duration is not varied, but rather the participant initiates a head turn after the onset of the stimulus, and the stimulus is gated off after the execution of 10 degrees of yaw rotation. So here duration may vary but total yaw does not. The statistics of the resulting behavioral head motions (latencies, accelerations, and durations) and how well the stimulated motion aligns with them is not discussed but should be. This would inform the authors' choice of which simulated durations to concentrate on. Given the 20 deg/s/s acceleration, the full 1000 ms would be required for 10 degrees of rotation.

- As the model now simulates the exact head movements, the duration discussion has become redundant. Statistics of the movements performed were added to the manuscript. Because motor noise does not scale with acceleration in our model, the acceleration does not affect performance in any way. Only duration and rotation size can affect the performance.

3) Given the closing paragraph (“The present analysis shows that this model can serve as a powerful framework ”…") I suggest adding some discussion of the possible application of the model to a variety of different phenomena that have arisen in previous and recent investigations of dynamic localization and of how it might need to be modified (in terms of: explicit weighting of binaural versus spectral cues; incorporating ILD or explicit dynamic cues; relaxing the assumption of a stationary source) to account for:

- substantial elimination of F/B errors with low-pass stimuli (no high-frequency spectral cues) and over ~100-ms constant duration regardless of yaw velocity

- authors' previous report (Front. Neurosci. 2023) that dynamic spectral cues are actually not important for small head turns

- Perrott & Noble's 1997 report of low-pass elevation perception with yaw movements

- Wallach illusion succeeding for low-pass stimuli but not for ones with potent real (e.g. wideband noise) or phantom (e.g. high-frequency narrow-band noise) spectral cues. Apparently the assumption of stationarity is abandoned for the latter stimulus types.

- These are indeed very interesting avenues for future applications of the Bayesian model, we have added a final paragraph to address these possibilities. To respond to each possible modification of the model:
  - Explicit weighting goes contrary to Bayesian modelling as the model itself will derive the weights of the cues depending on their relative uncertainty.
  - Cues to be taken into account by model can be chosen at will, assuming a relevant noise model can be derived from published experiments.
  - We have eliminated the spectral cues in the revision as there is evidence that dynamic spectral cues are not used for short head movements.
  - It is theoretically possible to adjust the assumption of a stationary source by including ´potential source movements in addition to potential source directions (this was done in Cox & Fischer, 2015 “Optimal prediction of moving sound source direction in the owl”). However, combining moving sources with a moving head would be computationally incredibly heavy, as it would increase the dimensionality of the problem significantly.

Line-by-line:

51-5“: "The proposed model utilises a ‘template-matching’ procedure which requires an acoustic template TA that the observed information is compared ”o.": Although the authors' model uses more sophisticated statistical techniques, a reference to Middlebrooks 1992 (Narrow‐band sound localization related to external ear acoustics. JASA, 92(5), 2607-2624.) would be appropriate as an originator of such "similarity over the sphere" -type models.

- Thanks for the pointer. We added this reference to the manuscript, and also included other localisation models that use this approach, such as Baumgartner et al. (2014), and Reijniers et al. (2014).

5“: "an acoustic template ”_A": wouldn’t it be clearer to refer to this as a _set_ of templates, one for each location in head-centred coordinates?

- Change made.

58-64: It makes sense to use only ITD, and not ILD, since ITD appears to dominate lateral-angle perception for such signals anechoically, but it would be good to mention this model design decision, and that fact that ILD would provide some source-spectrum independence that the monaural spectra lack.

- We would like to point out that because the spectral cues are considered for two ears, the cues presented to the model do contain frequency-dependent ILD information. As the model is an ideal observer, it takes all information, including the ILD cue, into account. It would be possible to add a broadband ILD as an extra cue (this was actually done in Barumerli et al., 2023, “A Bayesian model for human directional localization of broadband static sound sources”), though this would include the ILD cues twice, put a higher weight on the ILD cue, making the modelled observer non-ideal. Nevertheless, humans might still act as non-ideal observers, the extent of which was actually the goal of our work.

6“: "bandwidths (ERBs) following": missing text after "following"?

- Correct source was added

7“: "This stage simulates a simplified processing of the inner hair cell [”0]": although the authors of [20] may also have used such an IHC model, this does not seem like the best reference for this approach.

- Correct source was added

7“: "filterbank outputs were then summed over time": Not clear what this means.

- This was changed to: "Then, for each frequency channel, the root mean square of the signal was computed."

7“: "the ITD and the monaural spectral vectors for both ears are combined": this gives one vector element for ITD, but 64 elements for spectral information; how does this affect the relative influence of ITD and spectral cues?

- In optimal cue integration, the relative influence of each cue is defined by the noise on the signal, more specifically by the covariance matrix of the sensory input to the model. Changing the ITD noise, as was done in the discussion, shows that the model performs significantly worse when the ITD signal is less reliable.

14“: "marginalisation over all possible head orientations": does this literally mean _all_ possible orientations, or over the probability distribution of head orientations based on the accumulated sensorimotor evidence?

- Theoretically speaking, this would need to be done over all possible head rotations. However, in practical terms, the weights will indeed be much higher around the more probable head orientations. We also use this heuristic in our code to save on a significant amount of computational time. More specifically, we considered a range of 2*σ_H_ from the true head orientation, to contain about 95% of the noisy head observations.

19“: "Because this model computes the posterior recursively, as opposed to a single computation": is this really 'recursive' (which to me connotes a process that calls itself repeatedly and then "backs out" again), or merely "iterative" or cumulative? I would not call an IIR filter "recursive” just because past outputs are current inputs.

- To prevent confusion, we have changed our terminology to "cumulatively" here. However, the method used here is in fact referred to as “recursive Bayesian estimation” in the relevant literature.

226-22“: "A head-mounted display ... was used for the visual presentation of virtual reality (”R)": Describe the purpose of the VR. Were listeners' HRTFs measured while wearing the HMD, or can the authors comment on its acoustical or behavioural effect?

- It appears that the description in our methodology has caused some confusion, and we have now adjusted it accordingly. The head-mounted display (HMD) was used for three reasons: 1) The subject's head movements needed to be tracked, 2) The subject needed visual feedback on their orientation in space without seeing the loudspeakers 3) The subject needed visual feedback on their responses.

  Note that similar conditions could have been accomplished in a dark room, with a laser pointer and a separate head tracker, but the HMD solved all problems at once. Furthermore, localisation precision seems to be better with an HMD than in a dark room, likely because subjects get disoriented without any visual information about their orientation (Majdak et al, 201“, "3-D localization of virtual sound sources: Effects of visual environment, pointing method, and training.").

  Note also that the HRTFs were measured without the HMD, whereas the spectral cues during the experiment are indeed partially affected by an HMD. However, these differences were found to only minimally affect localisation performance (Poirier-Quinot & Lawless, 202“, "Impact of wearing a head-mounted display on localization accuracy of real sound sources" and Gupta et al., 201“, "Investigation of effect of VR/AR headgear on Head related transfer functions for natural listening").

  We now have added this reasoning to the Methods section.

244-24“: "Each trial used the same noise realisation, thus there was no variation of the spectral content between trials.": Not strictly true since each trial would involve a different duration cut-off of the noise waveform.

- We agree and adjusted the sentence accordingly.

272-273, Table 2: Mention in the caption that data from other studies are included here. Define "B-Static“, "M-Dynamic", etc. Why is B-Dynamic listed as 500 ms? Participants would have had a distribution of distributions, so is this representative?

- Thank you for the pointer - Indeed this was a typo. The abbreviations were improved and defined. The mean and SD values of the active condition durations are now provided correctly in the text, but are no longer reported in the table.

320: "The model localisation errors are reported for 100 ms. For this duration, the mean errors were similar to that of the behavioural data.": Provide more rationale for the choice of this comparison. For example, why would or would not a duration matching the listeners' mean duration be more apt? How best to compare durations between simulation and behavioural data when there must have been a considerable latency for listeners' initiation of movement?

- The model simulations were adjusted to match the exact movements made by the subjects in each trial, so there is no longer a difference between durations.

Fig 1, Fig 2, Fig 7: Specify the model duration in the captions and/or column labels.

- Reporting model duration here is no longer relevant, as it was identical to the experimental durations for consistency.

359-360: "responses may be pulled to certain directions due to compressions and expansions in the sensory representations of auditory (and visual) space [51]": A reference to Brimijoin 2018 (Angle-dependent distortions in the perceptual topology of acoustic space. Trends in Hearing, 22) would be appropriate here.

- Change made

367-368: "The biases did not change in the dynamic simulations, whereas the biases increased in the behavioural results.": Clarify wording to emphasize that the "change" or "increase" is for the dynamic situation relative to the static one.

- Change made

411-412: "However, previous studies have shown that the quantity and spatial distribution of reversal errors were highly subject-dependent.": Might it be good also to look at localization of low-pass stimuli (lacking high-frequency spectral cues) to inform the idea of a front/back spatial prior? I recall a paper by Simon Carlile showing a roughly 1/3 split between individuals consistently localizing such stimuli to the front, to the rear, or evenly mixed.

- Indeed, this serves as an interesting addition to the manuscript. Low-pass stimuli localisation showed a general bias towards the rear, although it was not the case for all subjects.

426-427: "The model results revealed that stimulus duration was a significant factor in the localisation task": Here it should be made explicit that duration is confounded with amount of rotation.

- Due to the change in the movement simulations, this problem solved itself.

436-438: "When head movements are allowed, a stimulus duration of approximately 100 ms seems to be required to provide a substantial benefit from the head movement [59]": Note that in that study, the 100-ms "threshold" applied to: stimuli lacking high-frequency spectral cues, widely different amounts of rotation depending on head-turn velocity, and to roughly constant velocity over the 100-ms period (i.e. not starting from rest).

- Thanks, we added this detail to the text.

439: "Fig 3 shows that the model reached a performance plateau around 500 ms": Point out that it is much earlier than 500 ms for QE rate, also mention how much rotation 500 ms corresponds to.

- A note was added for the QE rate. As discussed earlier, 500 ms correspond to 10 degrees, as do all other durations.

448-449: "Alternatively, it may be that humans only optimally integrate acoustic information up to 100 ms of listening": Adjust wording to make clear this is about localization; temporal integration for detection and loudness is 200-300 ms.

- As the time step size used in the new implementation was 100ms, we reformulated the manuscript accordingly: “Earlier work suggests that the auditory system can detect changes (ITD, ILD, spectrum) on a short time scale of about 5ms. However, the full integration window of acoustic information for sound localisation appears to be more in the range of 100-200ms (Zwislocki, 1969; Yabe, 1998).

454-455: "Assuming a possibly moving sound source would put a premium on forgetting old acoustic information as the state of the world would have changed in the meantime": This is also an argument for adding explicit, instantaneous dynamic cues to the model.

- We fully agree, and it would probably bring the model performance closer to human performance (higher lateral error while retaining QE improvement). However, we are not aware of physiological evidence for a dynamic ITD cue, which would make introducing this cue and its noise model in particular highly speculative. In any case, this is an important aspect in future modeling of this phenomenon.

456-457: "the lateral RMSE best matched the behavioural results at a single time step of 5 ms (5.5◦)." Were other time steps tried; how is it known that 5 ms was "best? Also, how is 5 ms equated with "5.5 degrees"?

- The section on stimulus duration was replaced with a section on different time steps. The base time step for the spatial analysis is now 100 ms, as most temporal integration windows were found to be around this duration.

457-458: "This suggests that the ITD information may not be integrated at all and is instead remeasured and reevaluated entirely at each time step." Am not following the argument here ... unpack?

- We removed this sentence as different durations were no longer tested. Instead, we tested the effect of the time step size. The results show that the time step has little effect on the modelled localisation performance.
